# An Asymptotic Analysis of Bivalent Monoclonal Antibody-Antigen Binding

**DOI:** 10.1007/s11538-025-01520-3

**Published:** 2025-08-26

**Authors:** Luke A. Heirene, Helen M. Byrne, James W. T. Yates, Eamonn A. Gaffney

**Affiliations:** 1 Mathematical Institute, University of Oxford, Andrew Wiles Building, Radcliffe Observatory Quarter, Woodstock Road, Oxford, OX2 6GG United Kingdom; 2 DMPK Modelling, DMPK, Preclinical Sciences, GSK, Gunnels Wood Road, Stevenage, SG1 2NY UK

**Keywords:** Antibodies, Immunotherapies, Asymptotic Analysis, ODE Model

## Abstract

Ligand-receptor interactions are fundamental to many biological processes. For example in antibody-based immunotherapies, the dynamics of an antibody binding with its target antigen directly influence the potency and efficacy of monoclonal antibody (mAb) therapies. In this paper, we present an asymptotic analysis of an ordinary differential equation (ODE) model of bivalent antibody-antigen binding in the context of mAb cancer therapies, highlighting the complexity associated with bivalency of the antibody. To understand what drives the complex temporal dynamics of bivalent antibody-antigen binding, we construct approximate solutions to the model equations at different timescales that are in good agreement with numerical simulations of the full model. We focus on two scenarios: one for which unbound antigens are abundant, and one for which they are scarce. We show how the dominant balance within the model equations changes between the two scenarios. Of particular importance to the potency and efficacy of mAb treatments are quantities such as antigen occupancy and bound antibody number. We use the results of our asymptotic analysis to estimate the long-time values of these quantities that could be combined with experimental data to facilitate parameter estimation.

## Introduction

Ligand-receptor interactions are ubiquitous in biology and medicine (Bongrand 1999). A ligand, often a small molecule or protein, binds specifically to a receptor protein on the surface of, or within, a cell (Teif 2005). Many processes within the body are initiated when a ligand binds to its receptor. For example, enzymes bind to substrates to catalyse biochemical reactions while hormones bind to their receptors to regulate physiology (Attie and Raines 1995). A key ligand-receptor interaction is an antibody binding to its target antigen (Goldberg 1952). Antibodies play an important role in adaptive immunity: they bind to harmful substances, such as toxins, to neutralise them (Forthal 2014). One type of antibody-based immunotherapy is based on monoclonal antibodies (mAbs). MAbs are used to treat many pathologies, including Alzheimer’s, autoimmune diseases, and cancer (Adams and Weiner 2005; Hafeez et al. 2018; Cummings 2023). For instance, cancer cells can suppress the immune response by engaging immune checkpoint receptors on the surface of lymphocytes, such as CD8 and CD4 T cells. MAbs, counteract this suppression by binding to checkpoint receptors and preventing their interaction with tumour ligands (Shiravand et al. 2022). Core to the mechanism of action of these inhibitors is the involvement of antibody binding to its target antigens on the cell surface. Therefore, increasing antigen occupancy in this context directly impacts the effectiveness of these therapies (Junker et al. 2021). Additionally, levels of bound antibody have been shown to influence the efficacy and potency of antibody effector functions such as antibody-dependent cellular cytotoxicity (ADCC) (Mazor et al. 2016; Junker et al. 2021). While many ligands are monovalent, the ability of mAbs to bind their target antigens is greatly enhanced by the fact that most mAbs have two antigen binding arms, i.e., they are bivalent (Vauquelin and Charlton 2013). The binding of multiple antigens decreases the effective dissociation of mAbs and cells leading to an apparent increase in binding strength, termed the “avidity effect" (Oostindie et al. 2022). Antibody-target antigen binding dynamics are important for the potency and efficacy of mAb therapies and are complicated by bivalency of the antibody. However, to the best of our knowledge, a detailed mathematical analysis of these binding dynamics in the context of cancer therapies and the avidity effect has not been completed. Mathematical modelling has been used to study ligand-receptor interactions in a variety of contexts (Perelson and DeLisi 1980; Klotz and Hunston 1984; Goldstein et al. 2004; Finlay et al. 2020; Dey et al. 2023). Ordinary differential equation (ODE) models have been used to study antibody-antigen interactions due to their simplicity (Kaufman and Jain 1992; Rhoden et al. 2016; Sengers et al. 2016; Heirene et al. 2025). Of these studies, (Perelson and DeLisi (1980)) has provided a detailed analysis of an ODE model of bivalent ligand receptor binding when the ligand is in excess. Here, we extend their analysis to the context of an antibody binding to antigens where the antibody concentration ranges over multiple orders of magnitude. Furthermore, due to antigen diffusion on the cell surface, binding of the second arm of the antibody is predicted to occur on a short timescale compared to the in solution binding of the first arm (Sengers et al. 2016; Heirene et al. 2025). Consequently, multiple timescales are present corresponding to the binding of the first and second arm respectively, and thus asymptotic analysis is a natural tool to characterise the binding dynamics. In previous work, we used mathematical modelling to determine how antibody-antigen interactions affect the avidity effect, equilibrium values of antigen occupancy and bound antibody numbers; these quantities are known to affect the potency and efficacy of immune checkpoint inhibitors and mAb effector functions, respectively (Heirene et al. 2025). We used global parameter sensitivity analysis to establish relationships between model parameters and antigen occupancy and bound antibody numbers. We found that the parameter dependencies are dose-dependent, with model outputs only sensitive to binding parameters, such as the off rate, at high antibody concentrations. However, in our previous work, we assumed the antibody-antigen interactions were at equilibrium. Here, we will instead consider antibody-antigen binding dynamics and use asymptotic analysis to address two main aims. The temporal dynamics of bivalent antibody-antigen binding is complex due to the reactivity of the second antibody binding arm. First, we use asymptotic analysis to elucidate the mechanisms that drive antibody-antigen binding on different timescales and across a range of antibody concentrations that commonly arise within in vitro experiments. We focus on in vitro experiments because they are widely used during the pre-clinical development of mAbs and can be more easily compared to the output of a simple mathematical model. Secondly, we use asymptotic analysis to identify those model parameters that determine the long-time behaviour of quantities that impact mAb potency and efficacy. In particular, we derive approximate expressions for antigen occupancy and bound antibody numbers. The remainder of the paper is organised as follows. In Section 2 we formulate and nondimensionalise our model of bivalent antibody-antigen binding. In Section 3 we show how the qualitative dynamics change with antibody concentration which we then analyse using perturbation methods in Section 4. The paper concludes in Section 5 where we summarise and discuss our results.

## Mathematical Model

**Fig. 1:**
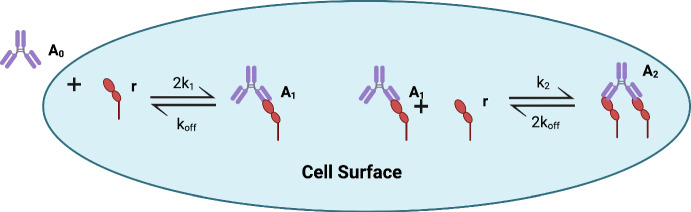
Schematic of a bivalent, monospecific antibody binding a target antigen on a cell surface. An unbound antibody, $A_0$, binds reversibly with a free target antigen, *r*, at a rate $k_{\text {on}}$, to form a monovalently bound antibody $A_{1}$ and dissociates at a rate $k_{\text {off}}$. $A_{1}$ binds a second free antigen at a rate $k_2$, to form a bivalently bound antibody $A_{2}$. A bivalently bound antibody can dissociate one of its bound arms away from the target antigen at a rate $k_{\text {off}}$. The factor of 2 appears in front of reaction rates where two antibody arms can undertake that reaction. In a slight abuse of notation, $A_{0}$ denotes a single unbound antibody but in Equations (1)-(4) it denotes the total number of unbound antibodies (similarly for all other variables). Created with Biorender.com.

In this section, we introduce a time-dependent mathematical model that describes the binding of a monospecific, bivalent antibody to target antigens on the surface of a single cell. We consider specifically the case of in vitro antibody binding studies. Our model is based on an existing model of bivalent ligand-receptor binding presented in Perelson and DeLisi (1980) and was first presented in Heirene et al. (2025). We apply the law of mass action to the reaction scheme shown in Figure 1 to derive a system of ODEs that describe the time evolution of the following dependent variables: the number of unbound target antigens, *r*(*t*); the number of unbound antibodies, $A_0(t)$; the number of monovalently bound antibodies, $A_1(t)$; and the number of bivalently bound antibodies, $A_2(t)$ (Voit et al. 2015). We assume that the system is well mixed and that target antigens are distributed uniformly over the cell surface. For simplicity, we neglect antigen internalisation. This is justified because, typically, the timescale of antigen internalisation (30 minutes to two hours in the case of mavrilimumab) is much slower than that of antibody binding (less than a second for high antibody concentrations) on the surface of a cell (Birtwistle and Kholodenko 2009; Vainshtein et al. 2014).Additionally, in what follows, we simulate the model for one hour, a standard duration for in vitro binding studies. We assume a free monospecific antibody binds one of its arms to a free target antigen to form a monovalently bound antibody-antigen complex. The monovalently bound antibody can either dissociate its antigen-bound arm or bind a second free antigen with its unbound arm to form a bivalently bound antibody. Each antigen-bound arm may then dissociate from its antigen. The rate at which one arm dissociates is assumed to be equal to, and independent of, the rate at which the other arm dissociates. Under the above assumptions and following the model development presented in Heirene et al. (2025), our mathematical model can be written as follows

1 $$\begin{aligned} \frac{\textrm{d}r}{\textrm{d}t}&= -2k_1rA_0 + k_{\text {off}}A_1 - k_2rA_1 + 2k_{\text {off}}A_2, \end{aligned}$$

2 $$\begin{aligned} \frac{\textrm{d}A_0}{\textrm{d}t}&= -2k_1rA_0 + k_{\text {off}}A_1, \end{aligned}$$

3 $$\begin{aligned} \frac{\textrm{d}A_1}{\textrm{d}t}&= 2k_1rA_0 - k_{\text {off}}A_1 - k_2rA_1 + 2k_{\text {off}}A_2, \end{aligned}$$

4 $$\begin{aligned} \frac{\textrm{d}A_2}{\textrm{d}t}&= k_2rA_1 - 2k_{\text {off}}A_2, \end{aligned}$$

where the reaction rates are defined and described in Figure 1. The factor of 2 that appears in the reaction terms $2k_1rA_0$ and $2k_{\text {off}}A_2$ arises when two antibody arms can undertake a reaction (e.g. an antibody in solution can bind either of its arms and, similarly, when it is bivalently bound either arm may dissociate). Equations (1)-(4) are closed by imposing the following initial conditions:

5 $$\begin{aligned} r(0)=r_{\text {tot}}, A_0(0)=A_{\text {tot}}, A_1(0)=0, A_2(0)=0. \end{aligned}$$

In Equation (5), we assume that all antigens are initially unbound, and we denote by $A_{\text {tot}}$ and $r_{\text {tot}}$ the total number of antibodies and target antigens respectively. The units of $A_{\text {tot}}$ and $r_{\text {tot}}$ are antibody number and antigen number respectively. By taking suitable linear combinations of Equations (1)-(4) and utilising Equation (5), it is straightforward to show that the total number of antibodies and antigens are conserved quantities within the system:

6 $$\begin{aligned} A_0 + A_1 + A_2&= A_{\text {tot}}, \end{aligned}$$

7 $$\begin{aligned} r + A_1 + 2A_2&= r_{\text {tot}}. \end{aligned}$$

We use Equations (6) and (7) to eliminate $A_0 =A_{\text {tot}} - A_1 - A_2$ and $r = r_{\text {tot}} -A_1 - 2A_2$ and, henceforth, focus on the following reduced system for $A_1(t)$ and $A_2(t)$:

8 $$\begin{aligned}&\frac{\textrm{d}A_1}{\textrm{d}t} = 2k_1(r_{\text {tot}}-A_1-2A_2)(A_{\text {tot}}-A_1-A_2) - k_{\text {off}}A_1 \nonumber \\&- k_2(r_{\text {tot}}-A_1-2A_2)A_1 + 2k_{\text {off}}A_2, \end{aligned}$$

9 $$\begin{aligned}&\frac{\textrm{d}A_2}{\textrm{d}t} = k_2(r_{\text {tot}}-A_1-2A_2)A_1 - 2k_{\text {off}}A_2, \end{aligned}$$

with

10 $$\begin{aligned} A_1(0)=A_2(0)=0. \end{aligned}$$

Before nondimensionalising our reduced model, we pause to estimate the model parameters.

### Model Parameter Estimates

Model parameter estimation was presented in Heirene et al. (2025) and is summarised here for completeness. Noting that assays within a reaction volume, $V_{\text {well}}$, (units: litres L), are used to estimate parameters, we estimate $A_{\text {tot}}$, the number of antibodies within the system, for a given experimental antibody concentration from

11 $$\begin{aligned} A_{\text {tot}} = A_{\text {init}}\sigma . \end{aligned}$$

Here, $A_{\text {init}}$ is the initial antibody concentration (units: molar concentration, M = mol/L), and $\sigma$ is a “concentration-to-antibody-number" conversion factor given by

12 $$\begin{aligned} \sigma = \frac{V_{\text {well}}Na}{T^0}, \end{aligned}$$

where $Na=6.02214 \times 10^{23}$ is Avogadro’s number (units: $\text {mol}^{-1}$) and $T^0$ is the number of target cells within the reaction volume. Equation (12) is normalised with respect to $T^0$, because we are focusing on binding to a single target cell. Estimates of $k_{\text {on}}$, the in-solution binding rate (units s$^{-1}$M$^{-1}$), are given for mAbs in the literature (Bostrom et al. 2011; Mazor et al. 2016). Here, however, we consider numbers of antibodies and antigens, rather than concentrations. Therefore, we need to rescale $k_{\text {on}}$ so its units are compatible with those used for $A_1$ and $A_2$:

13 $$\begin{aligned} k_1 = \frac{k_{\text {on}}}{\sigma }, \end{aligned}$$

where the units of $k_1$ are the number of antibodies per second. We assume that binding of the second arm of the antibody to antigens on the cell surface is limited by antigen diffusion (Sengers et al. 2016). As shown in our previous work (Heirene et al. 2025) this is because after an antibody binds a single antigen, it is unlikely that a second antigen will be close enough to bind the antibody’s second arm. Therefore, we assume that binding of the second arm is driven by the antigen’s ability to diffuse across the cell surface until it gets close enough to bind the antibody. From a consideration of first passage time processes (Heirene et al. 2025) and references therein as well as (Sengers et al. 2016), we suppose that this reaction occurs at rate $k_2$ where

14 $$\begin{aligned} k_2 = \frac{D}{4\pi (T_{\text {rad}})^2}. \end{aligned}$$

In Equation (14), *D* is the diffusion coefficient of the target antigen (units: $\text {m}^2\text {s}^{-1}$) and $T_{\text {rad}}$ is the radius of the target cell (units: metres). For reference, the model parameters and their interpretations are provided in Table 1.

**Table 1:** Model parameters associated with Equations (1)-(4)

Parameter	Definition	Estimated Value (units)	Source
$r_{\text {tot}}$	Target cell antigen number	$10^4 - 10^6$ (receptors per cell)	Mazor et al. (2016)
$k_{\text {on}}=\sigma k_1$	Antibody in solution binding rate	$10^5 - 6 \times 10^5$ (s$^{-1}$ M$^{-1}$)	Bostrom et al. (2011); Mazor et al. (2016)
$k_{\text {off}}$	Antibody dissociation rate	$10^{-4}-10^{-2}$ (s$^{-1}$)	Bostrom et al. (2011); Mazor et al. (2016)
$A_{\text {init}}$	Initial antibody concentration	$10^{-12} - 10^{-5}$ (M)	Pollard (2010)
$T^{0}$	Target cell number in assay	$2\times 10^5$ (cells)	Yu et al. (2017)
$V_{\text {well}}$	Assay reaction well volume	150 $(\mu$L)	Yu et al. (2017)
$T_{\text {rad}}$	Tumour cell radius	8 $(\mu$m)	Hosokawa et al. (2013)
*D*	Target antigen membrane diffusion coefficient	$10^{-15}-10^{-13}$ (m$^2$s$^{-1}$)	McCloskey and Poo (1986)
$k_2$	Diffusion limited second arm binding rate	$10^{-5} - 10^{-3}$ (s$^{-1}$)	Sengers et al. (2016)
$\sigma = V_{\text {well}} Na/T^0$	Concentration to protein number conversion factor	$4.5 \times 10^{14}$ (M$^{-1}$cell$^{-1}$)	
*Na*	Avogadro constant	$6.02214 \times 10^{232}$ (mol$^{-1}$)	

#### Nondimensionalisation

We nondimensionalise our model, by introducing the following scalings

15 $$\begin{aligned} A_0 = A_{\text {tot}}\hat{A_0}, A_1=r_{\text {tot}}\hat{A_1}, A_2 = r_{\text {tot}}\hat{A_2}, r=r_{\text {tot}}\hat{r}, t=\hat{\tau }/k_{\text {off}} . \end{aligned}$$

Dropping the hats for notational simplicity, we arrive at the following dimensionless equations

16 $$\begin{aligned} \frac{\textrm{d}A_1}{\textrm{d}\tau }&= 2\alpha _1 (1-A_1-2A_2)(\beta - A_1 - A_2) - A_1 - \alpha _2(1-A_1-2A_2)A_1 + 2A_2, \end{aligned}$$

17 $$\begin{aligned} \frac{\textrm{d}A_2}{\textrm{d}\tau }&= \alpha _2(1-A_1-2A_2)A_1 - 2A_2, \end{aligned}$$

with

18 $$\begin{aligned} A_1(0)= A_2(0) = 0. \end{aligned}$$

In Equations (16) and (17), we have introduced the following dimensionless parameter groupings

19 $$\begin{aligned} \alpha _1 = k_1r_{\text {tot}}/k_{\text {off}}, \alpha _2 = k_2r_{\text {tot}}/k_{\text {off}}, \beta = A_{\text {tot}}/r_{\text {tot}}. \end{aligned}$$

For reference, the nondimensional parameters are provided in Table 2. Upon nondimensionalisation, Equations (6) and (7) become

20 $$\begin{aligned} 1&= A_0 +\frac{1}{\beta }\bigg (A_1 + A_2 \biggr ), \end{aligned}$$

21 $$\begin{aligned} 1&= r + A_1 + 2A_2. \end{aligned}$$

These constraints state that the number of antibodies bound to the cell surface and antigens bound with antibody are bounded by the total number of antibodies within the system and the target antigen density respectively. We deduce further that antibodies will bind to the cell surface until either antibodies or free antigens run out. We note that the rate of binding of the second arm of the antibody on the cell surface is fast compared to the binding of the first arm ($\alpha _2 \gg \alpha _1)$. This drives the avidity effect where the increase in the number of bivalently bound antibody, $A_2$, contributes to an increase in binding strength because bivalently bound antibodies are less likely to dissociate. Physically, $\alpha _2 \gg \alpha _1$ is due to the proximity of free antigens to the antibody and the ability of antigens to diffuse across the cell surface. As such, we define a small parameter, $\epsilon$, as follows:

22 $$\begin{aligned} \epsilon = \sqrt{\frac{\alpha _1}{\alpha _2}}. \end{aligned}$$

There are certain regimes where the antigen diffuses slowly and the off rate is very fast such that the chance of an antibody binding a second antigen is unlikely. This situation however corresponds to a region of parameter space that is outside the bounds provided in Table 1 and also contradicts the aim of this analysis to study bivalent binding.

For $r_{\text {tot}}=10^5$, $k_{\text {on}}=10^5$ M$^{-1}$s$^{-1}$, $k_{\text {off}}=10^{-4}$ s$^{-1}$ and $k_2=10^{-5}$ s$^{-1}$, a typical set of parameter values, we obtain that $\alpha _1=\text {ord}(1)$ and $\alpha _2=\text {ord}(10^4)$ and so $\epsilon = \text {ord}(10^{-2})$. Here, the notation $X=\text {ord}(Y)$ means *X*/*Y* is strictly of order unity as $\epsilon \rightarrow 0$ (Hinch 1991). Making use of Equation (22), Equations (16) and (17) supply the full singular perturbation problem:

23 $$\begin{aligned} \frac{\textrm{d}A_1}{\textrm{d}\tau }&= 2\alpha _1 (1-A_1-2A_2)(\beta - A_1 - A_2) - A_1 - \frac{\alpha _1}{\epsilon ^2}(1-A_1-2A_2)A_1 + 2A_2, \end{aligned}$$

24 $$\begin{aligned} \frac{\textrm{d}A_2}{\textrm{d}\tau }&= \frac{\alpha _1}{\epsilon ^2}(1-A_1-2A_2)A_1 - 2A_2, \end{aligned}$$

While $\alpha _1=\text {ord}(1)$ corresponds to a standard set of parameter values, further justification of the relative size of $\alpha _1$ compared to other terms in Equations (23) and (24) is required to validate the asymptotic analysis included below. We provide this justification in appendix A.

**Table 2:** The dimensionless parameters $\alpha _1$ and $\alpha _2$ are constrained such that $\alpha _2 \gg \alpha _1$, so that the secondary binding is rapid compared to the primary binding. We take $\alpha _1=\text {ord}(1)$ in subsequent analysis

Parameter	Definition	Estimated Value
$\alpha _1 = \frac{k_1r_{\text {tot}}}{k_{\text {off}}}$	Nondimensional antibody in solution target binding rate	$4.4 \times 10^{-4}- 13.2$
$$\alpha _2 = \frac{k_2r_{\text {tot}}}{k_{\text {off}}}$$	Nondimensional diffusion limited second arm binding rate	$$1 - 10^6 $$
$\beta = \frac{A_{\text {tot}}}{r_\text {tot}}$	Ratio of Antibody to target receptor within the system	$5 \times 10^{-4} - 5 \times 10^5$

## Bivalent Model Dynamics

In the previous section, we introduced the parameter $\beta =A_{\text {tot}}/r_{\text {tot}}$, the ratio of total antibody to antigen number. With $r_{\text {tot}} = 10^5$, a typical antigen density for a tumour cell (Mazor et al. 2015), $\beta$ ranges over the following orders of magnitude as the antibody concentration $A_{\text {init}}$ varies over a range commonly used within in vitro experiments:

1. $$A_{\text {init}}= 10^{-11} \text { M} \Leftrightarrow \beta = \text {ord}(\epsilon )$$
2. $$A_{\text {init}}= 10^{-9} \text { M} \Leftrightarrow \beta = \text {ord}(1)$$
3. $$A_{\text {init}}= 10^{-7} \text { M}\Leftrightarrow \beta = \text {ord}(\epsilon ^{-1})$$
4. $$A_{\text {init}}= 10^{-5} \text { M}\Leftrightarrow \beta = \text {ord}(\epsilon ^{-2})$$

**Fig. 2:**
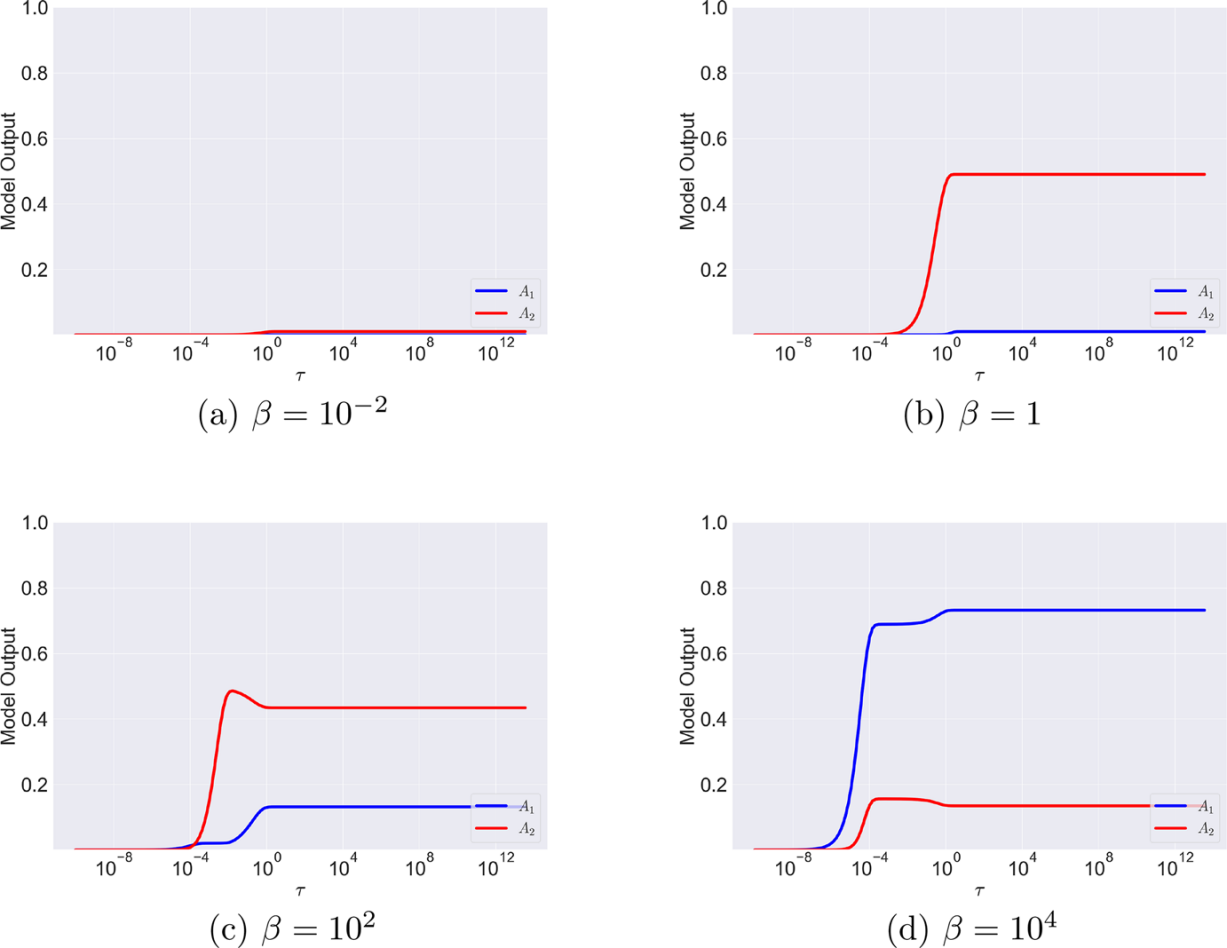
Numerical solutions of Equations (23) and (24) for different values of $\beta$ with $\alpha _1=1$, $\alpha _2 = 10^4$ and corresponding dimensional parameters $r_{\text {tot}}=10^5$, $k_{\text {on}}=10^5$ M$^{-1}$s$^{-1}$, $k_{\text {off}}=10^{-4}$ s$^{-1}$ and $k_2=10^{-5}$ s$^{-1}$. Initial condition of the system is given by Equation (18)

**Fig. 3:**
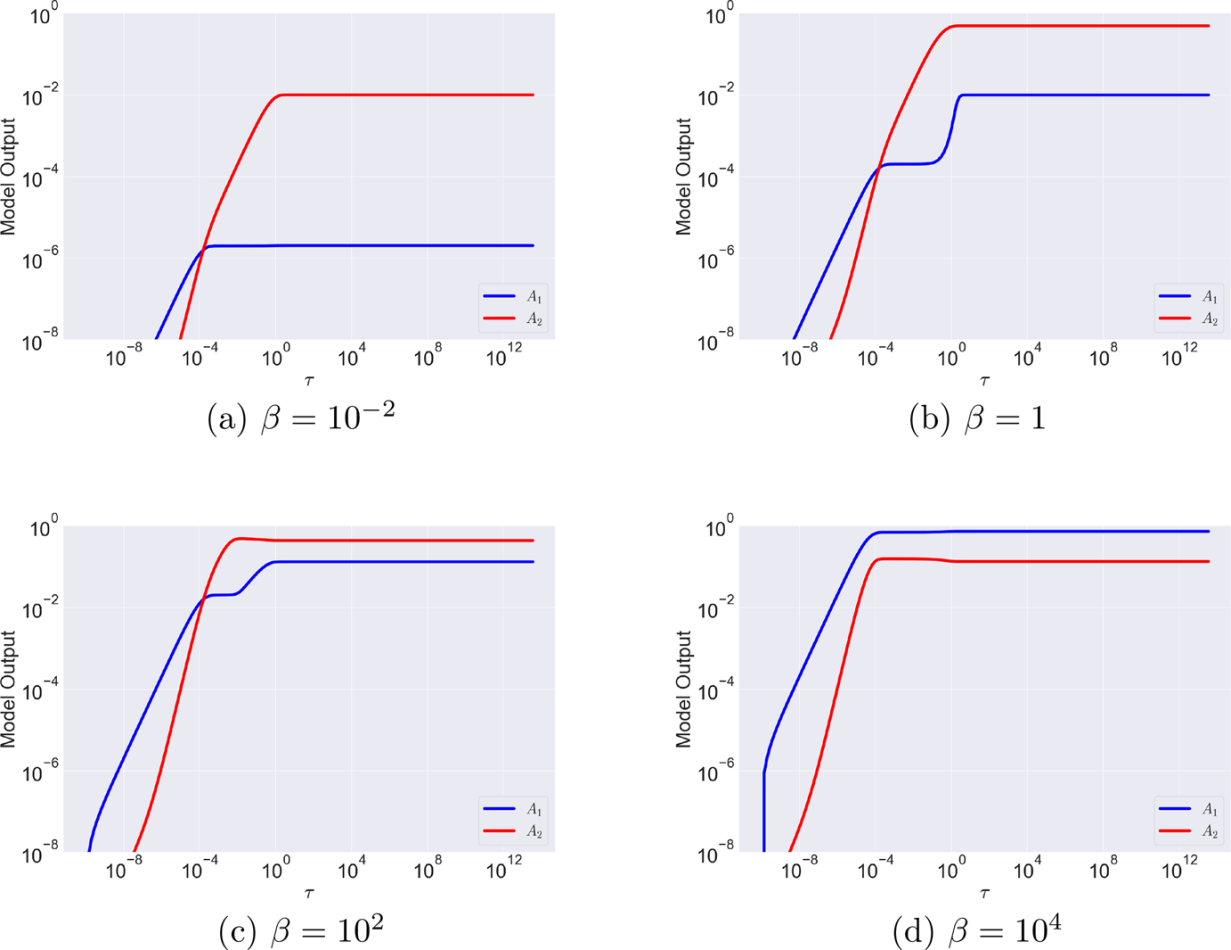
Model Dynamics plotted on a log scale from Equations (23) and (24) for different orders of magnitude of $\beta$ with $\alpha _1=1$, $\alpha _2 = 10^4$ and corresponding dimensional parameters $r_{\text {tot}}=10^5$, $k_{\text {on}}=10^5$ M$^{-1}$s$^{-1}$, $k_{\text {off}}=10^{-4}$ s$^{-1}$ and $k_2=10^{-5}$ s$^{-1}$. Initial condition of the system is $A_1(0)=A_2(0)=0$

Note that if the value of $r_{\text {tot}}$ were to change, the same magnitudes of $\beta$ can be achieved by adjusting $A_{\text {init}}$. The only condition we require for the analysis that follows is $\alpha _2 = k_2r_{\text {tot}}/k_{\text {off}} \gg \alpha _1$ to account for the avidity effect. In Figures 2 and 3, we present simulations of Equations (16) and (17) as $\beta$ varies. We plot the dynamics on a log scale in Figure 3 to observe the early time dynamics (we will more formally establish the duration of the early timescales in the asymptotic analysis that follows). We summarise the results below:

- Figures 2aand 3a($\beta = \text {ord}(\epsilon )$). Here, antigens are in excess since the number of antibodies is small. On a very fast timescale, there are more monovalently bound than bivalently bound antibodies (Figure 3a). As time progresses, the number of bivalently bound antibodies increases as the second arm binds. There are fewer monovalently bound antibodies because free antigens are in excess; once an antibody binds one arm, its second arm also easily binds with an available antigen. Since the number of antibodies is small, the total amount of binding is small, and $A_2$ reaches a maximum value of $A_2=0.01$.
- Figures 2band 3b($\beta = \text {ord}(1)$). Here, antibody and antigen levels are similar. Once again, at small times there is a short lag between monovalent and bivalent binding (Figure 3b). There are very few monovalently bound antibodies, but their numbers increase slightly at longer timescales as bivalently bound antibodies dissociate one of their arms. Competition for binding sites remains small because the magnitude of the reaction rate associated with the second binding event is large ($\alpha _2 \gg \alpha _1$) so, as for $\beta =\text {ord}(\epsilon )$, $A_2$ dominates $A_1$.
- Figure 2cand 3c($\beta = \text {ord}(\epsilon ^{-1})$). In this regime, antibody is in excess. Similar to $\beta =\text {ord}(\epsilon )$ and $\beta =\text {ord}(1)$, at early times there are more monovalently than bivalently bound antibodies. Despite increased competition for binding sites, since the reaction rate of the second binding event is much larger than that of the first binding event ($\alpha _2 \gg \alpha _1$), the surface reaction “out-competes" the in solution reaction and $A_2$ dominates as time increases. However, on a longer timescale, one arm of the bivalently bound complex dissociates and the free antigen is quickly bound by a free antibody. Consequently, the number of bivalently bound antibodies decreases and the number of monovalently bound antibodies increases but there are still more bivalently bound than monovalently bound antibodies.
- Figure 2d3d($\beta = \text {ord}(\epsilon ^{-2})$). Here, a large number of antibodies compete for a comparatively small number of free antigens, driving an effective increase in the rate at which the first binding event takes place so that it out-competes the fast surface reaction that forms $A_2$, and $A_1$ dominates. As seen with smaller values of $\beta$, at longer timescales dissociation events drive another increase in $A_1$.

## Asymptotic Analysis of Bivalent Model

Here, we construct approximate solutions to Equations (23) and (24) across different timescales. In the previous section, to understand how the dynamics develop, we considered four values of $\beta$ that commonly arise within in vitro experiments. However,for the asymptotic analysis of Equations (23) and (24), there are only two distinguished limits to consider:

1. Antibody levels are sufficiently low that the effective monovalent binding rate is smaller than the bivalent binding rate; that is, $\text {ord}(\alpha _1\beta ) \ll \text {ord}(\alpha _2)$.
2. Antibody levels are sufficiently large that the monovalent and bivalent binding reaction rates are of the same magnitude,that is, $\text {ord}(\alpha _1\beta ) = \text {ord}(\alpha _2)$.

For the simulations present in Section 3, case one applies when $\beta =\text {ord}(\epsilon )$, $\beta =\text {ord}(1)$ and $\beta =\text {ord}(\epsilon ^{-1})$ while case two applies when $\beta =\text {ord}(\epsilon ^{-2})$. We analyse these cases separately in what follows. Technically there is also the case $\text {ord}(\alpha _1\beta ) > \text {ord}(\alpha _2)$. However, this requires $\beta \gg \alpha _1/\alpha _2$, corresponding to an unrealistically large antibody concentration and so we do not consider this case.

### Case 1: $\text {ord}(\alpha _1\beta ) < \text {ord}(\alpha _2)$

While this case holds for all values of $\beta < \text {ord}(\epsilon ^{-2}$), we focus on the specific case $\beta =\text {ord}(1)$, $\alpha _1=\mathcal {O}(1)$. The structure of the analysis remains consistent for $\beta =\text {ord}(\epsilon )$ and $\beta =\text {ord}(\epsilon ^{-1})$, with variations arising only in the scalings of $A_1$ and $A_2$ and $\tau$ required to satisfy the dominant balance. We will highlight these differences where relevant.

#### Initial Transients and Fast Timescales, $\beta <1/2$

First, we will consider the fast timescales and initial transients of the system. In order to do this, we balance the large avidity term ($\alpha _1A_1/\epsilon ^2$) with the derivatives by rescaling $A_1 = \epsilon ^2 \bar{A}_1$, $A_2 = \epsilon ^2 \bar{A}_2$ and $\tau = \epsilon ^2 \bar{\tau }$ and consider the following asymptotic expansions

25 $$\begin{aligned} \bar{A}_1&= \bar{A}_{1,0} + \epsilon \bar{A}_{1,1} + \cdots , \end{aligned}$$

26 $$\begin{aligned} \bar{A}_2&= \bar{A}_{2,0} + \epsilon \bar{A}_{2,1} + \cdots . \end{aligned}$$

Substituting Equations (25) and (26) into Equations (23) and (24) and collecting leading order terms we obtain

27 $$\begin{aligned} \frac{\textrm{d}\bar{A}_{1,0}}{\textrm{d}\bar{\tau }}&= 2\alpha _1\beta - \bar{A}_{1,0}, \end{aligned}$$

28 $$\begin{aligned} \frac{\textrm{d}\bar{A}_{2,0}}{\textrm{d}\bar{\tau }}&= \bar{A}_{1,0}. \end{aligned}$$

Using the initial conditions $\bar{A}_{1,0}=\bar{A}_{2,0}=0$, we arrive at the solutions

29 $$\begin{aligned} \bar{A}_{1,0}&= 2\alpha _1\beta (1 - e^{-\bar{\tau }}), \end{aligned}$$

30 $$\begin{aligned} \bar{A}_{2,0}&= 2\alpha _1\beta (\bar{\tau } + e^{-\bar{\tau }} -1) . \end{aligned}$$

In order to understand the initial transients of the system, we expand Equations (29) and (30) around $\bar{\tau }=0$ and obtain

31 $$\begin{aligned} \bar{A}_{1,0}&\approx 2\alpha _1\beta \bar{\tau } + \mathcal {O}(\bar{\tau }^2), \end{aligned}$$

32 $$\begin{aligned} \bar{A}_{2,0}&\approx \mathcal {O}(\bar{\tau }^2). \end{aligned}$$

This behaviour describes the the initial transients during which antibodies bind monovalently to the cell surface, but have not yet engaged their second arm. This can be seen in Figure 3 where there is a small increase in $A_1$, before $A_2$. Equation (31) is valid provided $\bar{\tau } \ll \text {ord}(1)$ (equivalently $\tau \ll \text {ord}(\epsilon ^2))$. When $\bar{\tau } \approx \text {ord}(1)$, the dynamics are governed by Equations (29) and (30) which correspond to a regime during which antibodies bind monovalently to the cell surface, while also engaging their second arm to form bivalently bound complexes. For $\bar{\tau }\gg 1$ noting that $\alpha _1=\text {ord}(1)$ we observe from Equations (29) and (30), that $A_1 \sim \text {ord}( \epsilon ^2\beta )$ and $A_2 \sim \text {ord}(\epsilon ^2\beta \bar{\tau })$, respectively. We conclude that with $\bar{\tau }\gg 1$, but still $\tau \ll 1$, the magnitude of $A_1$ is small compared to $A_2$ which increases with $\bar{\tau }$. The dominance of $A_2$ arises due to the availability of free antigen and the rapid rate of the second binding event.

#### Long Timescale Behaviour

Once $A_2 \sim \text {ord}(1)$, the relative balance of terms in Equations (23) and (24) shifts as $A_2$ becomes much larger than $A_1$. According to Equation (30), this transition occurs when $\beta \bar{\tau } \approx \text {ord}(\epsilon ^{-2})$, indicating a dependence on the value of $\beta$. For $\beta =\text {ord}(1)$, the appropriate timescale is $\tau \approx \text {ord}(1)$, whereas for $\beta =\text {ord}(\epsilon ^{-1})$, the appropriate timescale would be $\tau \approx \text {ord}(\epsilon )$. This scaling behaviour is expected: as the number of antibodies increases (i.e., larger $\beta$), more can bind to the cell and subsequently cross-link to form bivalently bound complexes and so it takes less time for $A_2$ to become $\text {ord}(1)$. To capture this for the case $\beta =\text {ord}(1)$, we scale $A_1 = \epsilon ^2 \bar{A}_1$ and $A_2, \tau \sim \text {ord}(1)$. Substituting these scalings into Equations (23) and (24) and collecting leading order terms we obtain

33 $$\begin{aligned}&0 = 2\alpha _1(\beta -A_2)(1-2A_2) - \alpha _1\bar{A}_1(1-2A_2) + 2A_2, \end{aligned}$$

34 $$\begin{aligned}&\frac{\textrm{d}A_2}{\textrm{d}\tau } = \alpha _1\bar{A}_1(1-2A_2) - 2A_2. \end{aligned}$$

Equation (33) supplies

35 $$\begin{aligned} \bar{A}_1 = \frac{2\alpha _1(\beta -A_2)(1-2A_2) + 2A_2}{\alpha _1(1-2A_2)}. \end{aligned}$$

Furthermore, combining Equations (33) and (34) gives

36 $$\begin{aligned} \frac{\textrm{d}A_2}{\textrm{d}\tau } = 2\alpha _1(\beta -A_2)(1-2A_2), \end{aligned}$$

with solution when $\beta \ne 1/2$:

37 $$\begin{aligned} A_2^{\text {out}} = \frac{\beta (e^{2(2\beta - 1)\alpha _1 \tau } - 1)}{2\beta e^{2(2\beta - 1)\alpha _1 \tau } - 1}\rightarrow {\left\{ \begin{array}{ll} \frac{1}{2} \text { as } \tau \rightarrow \infty \text { if } \beta > \frac{1}{2} \\ \beta \text { as } \tau \rightarrow \infty \text { if } \beta < \frac{1}{2} \end{array}\right. } \biggr \}=\text {min}\biggl (\frac{1}{2}, \beta \biggr ) \end{aligned}$$

When $\beta > 1/2$, there are enough antibodies to bind all antigens. As a result, antigens become saturated with bivalently bound antibody as $\tau \rightarrow \infty$. When $\beta < 1/2$, there are more antigens than antibody binding arms. Hence, all antibodies bind until there are no free antibodies. When $\beta =1/2$, Equation (36) supplies

38 $$\begin{aligned} A_2^{\text {out}} = \frac{\alpha _1 \tau }{2\alpha _1\tau +1} \rightarrow \frac{1}{2} \text { as } \tau \rightarrow \infty . \end{aligned}$$

Constructing composite solutions with the estimates of $A_1$ and $A_2$ we have obtained so far along with an intermediate variable we arrive at

39 $$\begin{aligned} A_1&= \epsilon ^2\biggl [2\alpha _1\beta (1-e^{-\tau /\epsilon ^2}) + \biggl (\frac{2\alpha _1(\beta -A_2^{\text {out}})(1-2A_2^{\text {out}}) + 2A_2^{\text {out}}}{\alpha _1(1-2A_2^{\text {out}})}\biggr ) - 2\beta \alpha _1\biggr ], \end{aligned}$$

40 $$\begin{aligned} A_2&= \epsilon ^22\alpha _1\beta \biggl (\frac{\tau }{\epsilon ^2} + e^{-\tau /\epsilon ^2}-1\biggr ) + A_2^{\text {out}} - 2\beta \alpha _1\tau , \end{aligned}$$

where $A_2^{\text {out}}$ is given by Equation (37) or (38) depending on the value of $\beta$ and noting $\alpha _1=1$. Equations (39) and (40) would be slightly different for other values of $\beta$ where the timescales and scalings of $A_1$ and $A_2$ in the separate regions differ as we have outlined through the analysis so far. The analysis up to this point sufficiently describes the dynamics for $\beta = \text {ord}(1), <1/2$, $|\beta -1/2| \gg \epsilon ^2$. In all cases, the long-time values of $A_1$ and $A_2$ can be defined from Equations (35) and (37). However, for $\beta \ge 1/2$ additional analysis is required since Equations (35) and (39) break as $A_2\rightarrow 1/2$, which we outline below.

#### Behaviour as Availability of Free Antigen Becomes Small, $\beta \ge 1/2$

From Equation (35), we see that the asymptotic expansion for $A_1=\text {ord}(\epsilon ^2)$ breaks down as $A_2\rightarrow 1/2$. Observing Equation (24) with $A_1=\text {ord}(\epsilon ^2)$, as $A_2\rightarrow 1/2$ the dominant balance within the equations changes. Physically, this corresponds to the number of free antigen becoming very small such that binding is as likely as a dissociation event. As a result, we require a new scaling to understand the system dynamics within this regime. First, we consider a general case for which $1/2\le \beta \ll \epsilon ^{-2}$ and define $\epsilon ^d\bar{\beta }=\beta -1/2$, so the closer $\beta$ is to 1/2 the larger the value of *d*. As we need to analyse the behaviour as $A_2\rightarrow 1/2$, let us also define

41 $$\begin{aligned} A_2 = \frac{1}{2} - \lambda . \end{aligned}$$

In what follows we consider the scalings $\lambda = \epsilon ^a \hat{\lambda }$, $A_1 = \epsilon ^b\hat{A}_1$ with $a,b >0$ where appropriate values of *a* and *b* need to be chosen. Expressing Equations (23) and (24) in terms of $A_1$ and $\lambda$ we have

42 $$\begin{aligned} \epsilon ^b \frac{\textrm{d}\hat{A}_1}{\textrm{d}\tau }&= 2\alpha _1\epsilon ^b(\epsilon ^d\bar{\beta } - \epsilon ^b\hat{A}_1 + \epsilon ^a\hat{\lambda })(2\epsilon ^{a-b}\hat{\lambda } - \hat{A_1}) - \epsilon ^b\hat{A}_1 \nonumber \\&- \alpha _1\epsilon ^{2(b-1)}\hat{A}_1(2\epsilon ^{a-b}\hat{\lambda } - \hat{A}_1) + 1 - 2\epsilon ^a\hat{\lambda }, \end{aligned}$$

43 $$\begin{aligned} \epsilon ^a \frac{\textrm{d}\hat{\lambda }}{\textrm{d}\tau }&= - \alpha _1\epsilon ^{2(b-1)}\hat{A}_1(2\epsilon ^{a-b}\hat{\lambda } - \hat{A}_1) + 1 - 2\epsilon ^a\hat{\lambda }, \end{aligned}$$

As mentioned previously, as $A_2 \rightarrow 1/2$, the number of free antigens becomes small. Therefore, the dominant balances are now between each of the binding terms and their respective dissociation terms. In Equation (43), the dominant balance is achieved by equating the first and third terms on the right-hand-side of the equation which yields

44 $$\begin{aligned} a+b=2. \end{aligned}$$

The dominant balance in Equation (42) is more subtle, owing to the potential variability in $\bar{\beta }$. When $\bar{\beta } \ne 0$, the dominant balance depends on the value of *d* which we outline below:

- $d<2/3$: The balance is between the $\bar{\beta }\hat{\lambda }$ and $-\hat{A}_1$. Using Equation (44), this gives
  45 $$\begin{aligned} a=1-\frac{d}{2}, \hspace{2mm} b=1 + \frac{d}{2}. \end{aligned}$$
- $d>2/3$: The balance is between the $\hat{\lambda }^2$ term with the $-\hat{A}_1$ giving
  46 $$\begin{aligned} a=\frac{2}{3}, \hspace{2mm} b=\frac{4}{3}. \end{aligned}$$
- $d=2/3$: Now the balance involves all terms from the cases above with $a=2/3, b=4/3$.

Regardless of the details of the dominant balance the solution method remains the same. For example, when $\bar{\beta }=\text {ord}(\epsilon )$, we have $a=2/3, b=4/3$. Setting $\textrm{d}/\textrm{d}\tau =0$ in Equation (43) we deduce that the system evolves to an equation for which, at leading order

47 $$\begin{aligned} \hat{\lambda }=\frac{1}{2\alpha _1\hat{A}_1}. \end{aligned}$$

Next, subtracting Equation (43) from Equation (42) with $\textrm{d}/\textrm{d}\tau =0$ and dividing through by $\epsilon ^{4/3}$ we obtain the following relationship at leading order

48 $$\begin{aligned} 0 = 4\alpha _1\hat{\lambda }^2 - \hat{A}_1 + \mathcal {O}(\epsilon ^{1/3}), \end{aligned}$$

obtaining

49 $$\begin{aligned} \hat{A}_1 = 4\alpha _1\hat{\lambda }^2. \end{aligned}$$

Combining Equations (47) and (49) we arrive at

50 $$\begin{aligned} \hat{A}_1 = \frac{1}{\alpha _1^{1/3}}, \hspace{2mm} \hat{\lambda } = \frac{1}{2\alpha _1^{2/3}}. \end{aligned}$$

More generally, Equation (50) is valid when $d>2/3$, i.e, values of $\beta$ close to 1/2. We will simply state the results for the other cases. If $\bar{\beta }$ scales with $\epsilon ^{1/3}$ so that $d=1/3, a=5/6, b=7/6$ or more generally when $\bar{\beta }$ is of a scale to ensure it is the dominant balance, we obtain the following expressions for $\hat{\lambda }$ and $\hat{A}_1$

51 $$\begin{aligned} \hat{A}_1 = 2\sqrt{\bar{\beta }}, \hspace{2mm} \hat{\lambda }&= \frac{1}{2\alpha _1\sqrt{\bar{\beta }}}. \end{aligned}$$

Equation (50) is valid for $\{d \in (-2,2/3) | d\ne 0\}$. We treat the special case $d=0$ and $a=b=1$ separately as additional terms appear in Equations (42) and (43) at leading order. In this case, the expressions for $\hat{\lambda }$ and $\hat{A}_1$ are

52 $$\begin{aligned} \hat{A}_1 = 2\bar{\beta }, \hspace{2mm} \hat{\lambda } = \frac{1 + 2\alpha _1\bar{\beta }}{2\alpha _1}. \end{aligned}$$

For the edge case $d=2/3, a=2/3, b=4/3$, we have that $\hat{\lambda }=1/(2\hat{A}_1)$ while $\hat{A}_1$ satisfies

53 $$\begin{aligned} \alpha _1\hat{A}_1^3 - 2\alpha _1\bar{\beta }\hat{A}_1 - 1=0 \end{aligned}$$

**Fig. 4:**
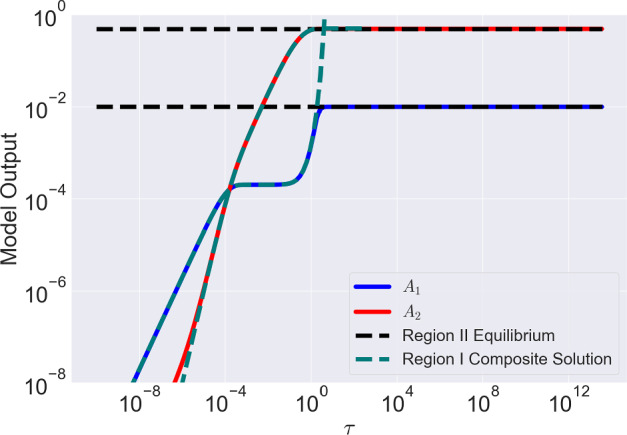
Comparison between the numerical solutions of Equations (23) and (24), and estimates obtained through asymptotic analysis for $\beta =1, \alpha _1=1$ and corresponding dimensional parameters $r_{\text {tot}}=10^5$, $k_{\text {on}}=10^5$ M$^{-1}$s$^{-1}$, $k_{\text {off}}=10^{-4}$ s$^{-1}$ and $k_2=10^{-5}$ s$^{-1}$. We also note that $\epsilon =10^{-2}$ The composite solutions of the Region I estimates are given by Equations (39) and (40) while the Region II asymptotes are given by Equations (52) and (41). We cut off the y-scale at $10^{-8}$ due to the solutions tending to 0 at $\tau =0$ which causes visualisation problems when plotting on a log scale

For a comparison of the results of the asymptotic analysis when $\bar{\beta }=\text {ord}(1)$ with the solutions of the full equations see Figure 4.

To summarise, the results of this analysis with $\beta \ge 1/2, \text {ord}(\alpha _1\beta )<\text {ord}(\alpha _2)$, describe the existence of two regions with the dynamics

- Region I: Here, the number of unbound antigens is large enough such that $1-A_1-2A_2\approx \text {ord}(1)$. This region also includes the initial transients of the system.
- Region II: As antibodies continue to bind free antigen, the number of available antigens decreases until antigens are almost saturated with antibody. This shift alters the dominant balance in the model equations: terms associated with antibody antigen dissociation become leading order. To capture the system’s behaviour in this regime, a new scaling of the variables is necessary. Consequently, the dynamics of $A_1$ and $A_2$ are fundamentally different in Region II. Within Region II, the system relaxes to a new long time equilibrium that depends on the value of $\beta$ as detailed in Section 4.1.3.

**Fig. 5:**
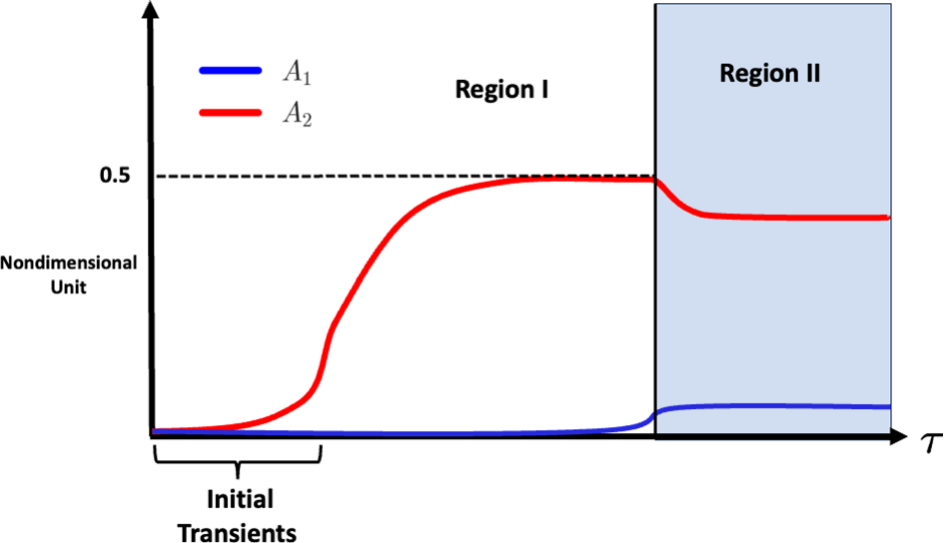
Figure depicting Regions I and II within the dynamics valid when $\beta \ge 1/2$. After initial transients, $A_2$ approaches a quasi-steady equilibrium defined by Equation (37) or (38). Region I is where there are many free antigens available while Region II is where the number of free antigens is small such that the size of terms in Equations (23) and (24) change and a new set of equations need to be derived to develop a simple approximation of the dynamics. The curves displayed in this Figure and Figure 4 are among the possibilities as parameters vary but the general behaviour remains the same

A visual depiction of Regions I and II is presented in Figure 5.

#### Expressions for Antigen Occupancy and Bound Antibody Number

A goal of our analysis is to derive analytic expressions for the long time values of antigen occupancy and bound antibody number, key correlates of mAb treatment effectiveness. We define

54 $$\begin{aligned} A_{occ}&:= A_1 + 2A_2, \end{aligned}$$

55 $$\begin{aligned} A_{bound}&:= A_1 + A_2, \end{aligned}$$

where $A_{occ}$ and $A_{bound}$ are antigen occupancy and bound antibody number respectively. When $\beta <1/2$, Equation (37) supplies

56 $$\begin{aligned} A_{occ}&\approx 2A_2 = 2\beta , \end{aligned}$$

57 $$\begin{aligned} A_{bound}&\approx A_2 = \beta . \end{aligned}$$

For this range of $\beta$ values, the number of monovalently bound antibodies is negligible; nearly all bound antibodies engage both arms. Additionally, because the number of antibodies is lower than the number of available antigens, binding proceeds until all antibodies are depleted and $A_{bound}\approx \beta$. In Table 3 within the discussion we state the long time values of $A_{occ}$ and $A_{bound}$ obtained from the analysis in Section 4.1.3. We omit the special case $\beta -1/2=\text {ord}(\epsilon ^{2/3})$ since $A_1$ has to be solved for numerically. From Table 3, we see that at leading order $A_{occ}\approx 1$ i.e., antigens are fully occupied. This is due to each antibody being able to bind two antigens each with ease. The behaviour of $A_{bound}$ in Table 3 is not as clear aside from $A_{bound}=1/2$ at leading order. Using $\beta -1/2=\text {ord}(1)$ as an example and noting that $\epsilon = \sqrt{\alpha _1/\alpha _2}$, we have

58 $$\begin{aligned} A_{bound} \approx \frac{1}{2} + \frac{1}{\sqrt{\alpha _2}}\biggl [\sqrt{\alpha _1}\biggl (\beta -\frac{1}{2}\biggr ) - \frac{1}{2\sqrt{\alpha _1}}\biggr ] . \end{aligned}$$

Equation (58) indicates that increasing the monovalent binding rate and antibody dose as measured by $\alpha _1$ and $\beta$ respectively, can enhance the number of bound antibodies. Interestingly, the equation also suggests that higher avidity, quantified by $\alpha _2$, may reduce the number of bound antibodies. This effect likely arises because bivalent binding removes two antigens per antibody, thereby limiting the number of binding opportunities for other antibodies in solution and ultimately decreasing the total number of bound antibodies. However, in the mentioned parameter regimes the resulting change in $\beta$ is predicted to be small.

### Case 2: $\beta = \text {ord}(\alpha _2/\alpha _1) = \text {ord}(1/\epsilon ^2)$

When $\beta = \text {ord}(\epsilon ^{-2})$, we have

59 $$\begin{aligned} \frac{\textrm{d}A_1}{\textrm{d}\tau }&= 2\alpha _1\biggl (\frac{\hat{\beta }}{\epsilon ^2} - A_1 - A_2\biggr )(1-A_1-2A_2) - A_1 - \frac{\alpha _1A_1}{\epsilon ^2}(1-A_1-2A_2) + 2A_2, \end{aligned}$$

60 $$\begin{aligned} \frac{\textrm{d}A_2}{\textrm{d}\tau }&= \frac{\alpha _1A_1}{\epsilon ^2}(1-A_1-2A_2) - 2A_2. \end{aligned}$$

Here, $\beta$ is sufficiently large that the effective rates of monovalent and bivalent binding are similar in magnitude. As a result, a new dominant balance arises and the analysis differs from case 1, and is detailed below.

#### Region I Solution

Figure 3d shows that the sharp increase in monovalent binding at early times is followed by bivalent binding. In contrast to case 1, during this short timescale, the number of monovalent antibodies is larger than the number of bivalent antibodies. Accordingly, we fix $A_1 = \epsilon \bar{A}_1$, $A_2 = \epsilon ^2 \bar{A}_2$ and $\tau = \epsilon ^3\bar{\tau }$ and Equations (59) and (60) supply at leading order

61 $$\begin{aligned} \frac{\textrm{d}\bar{A}_1}{\textrm{d}\bar{\tau }}&= 2\alpha _1 \hat{\beta },\end{aligned}$$

62 $$\begin{aligned} \frac{\textrm{d}\bar{A}_2}{\textrm{d}\bar{\tau }}&= \alpha _1\bar{A}_1, \end{aligned}$$

with solutions

63 $$\begin{aligned} \bar{A}_1&= 2\alpha _1\hat{\beta }\bar{\tau }, \end{aligned}$$

64 $$\begin{aligned} \bar{A}_2&= \alpha _1^2\hat{\beta }\bar{\tau }^2. \end{aligned}$$

Thus, both $A_1$ and $A_2$ increase at rates proportional to the effective monovalent binding rate, $\alpha _1 \beta$ (with an extra factor of $\alpha _1$ for $\bar{A}_2$ due to avidity). With $\beta =\text {ord}(\epsilon ^{-2})$ $A_1$ and $A_2$ grow rapidly, and we require a new scaling when $\tau \approx \mathcal {O}(\epsilon ^2)$. As such, we assume $A_1$ and $A_2$ are $\text {ord}(1)$ and rescale $\tau = \epsilon ^2\tau ^*$. Substituting these scalings into Equations (59) and (60) we obtain at leading order

65 $$\begin{aligned} \frac{\textrm{d}A_1}{\textrm{d}\tau ^*}&= 2\alpha _1\hat{\beta }(1-A_1-2A_2) - \alpha _1A_1(1-A_1-2A_2), \end{aligned}$$

66 $$\begin{aligned} \frac{\textrm{d}A_2}{\textrm{d}\tau ^*}&= \alpha _1A_1(1-A_1-2A_2). \end{aligned}$$

We simplify the analysis of Equations (65) and (66), by defining

67 $$\begin{aligned} p := 2(1-A_1-2A_2). \end{aligned}$$

Recasting Equations (65) and (66) in terms of *p* and $A_1$ supplies

68 $$\begin{aligned} \frac{\textrm{d}A_1}{\textrm{d}\tau ^*}&= p\biggl (\alpha _1\hat{\beta } - \frac{A_1}{2}\biggr ), \end{aligned}$$

69 $$\begin{aligned} \frac{\textrm{d}p}{\textrm{d}\tau ^*}&= -p\biggl (2\alpha \hat{\beta } + \frac{A_1}{2}\biggr ), \end{aligned}$$

where $p(0) = 2$. Using Equations (68) and (69), it can be shown $p\rightarrow 0$ and $A_1\rightarrow A_1^*$ constant, as $\tau ^* \rightarrow \infty$ (for details, see Appendix B). The latter may also be rapidly deduced by noting $A_1\ge 0$, and hence that $\textrm{d}p/\textrm{d}\tau ^* \le 0$ in Equation (69) so *p* decays at an exponential rate of $2\alpha \hat{\beta }$ or faster. Hence, we have

70 $$\begin{aligned} A_1+2A_2 \rightarrow 1 \text { as } \tau ^* \rightarrow \infty , \end{aligned}$$

so at long times all antigens become bound with antibody. When $1-A_1-2A_2\approx \text {ord}(\epsilon ^2)$ Equations (68) and (69) are no longer valid and we enter region II.

#### Region II Solution

In region II, $p\approx \text {ord}(\epsilon ^2)$ and $A_1\rightarrow A_1^*$. To capture the region II dynamics, we assume $\tau \approx \text {ord}(1)$ and recast Equations (59) and (60) in terms of $A_1$ and *p*:

71 $$\begin{aligned} \frac{\textrm{d}A_1}{\textrm{d}\tau }&= \frac{\alpha _1p}{\epsilon ^2}\biggl (\hat{\beta } - \frac{A_1}{2}\biggr ) +\frac{\alpha _1p}{2}\biggl (\frac{p}{2} - A_1 -1\biggr ) -2A_1 - \frac{p}{2} +1, \end{aligned}$$

72 $$\begin{aligned} \frac{\textrm{d}p}{\textrm{d}\tau }&= -\frac{\alpha _1p}{\epsilon ^2}\biggl (2\hat{\beta } + A_1\biggr ) -\frac{\alpha _1p}{2}\biggl (\frac{p}{2} - A_1 -1\biggr ) +2 - p. \end{aligned}$$

We describe the behaviour when the number of free antigens becomes small by rescaling $p = \epsilon ^2\kappa$. Recasting Equations (71) and (72) in terms of $A_1$ and $\kappa$ with $\textrm{d}/\textrm{d}\tau =0$ since we are interested in the long-time equilibrium behaviour of the system, we obtain the following leading order expressions for the steady state values of $\kappa$ and $A_1$:

73 $$\begin{aligned}&\kappa = \frac{2}{\alpha _1(2\hat{\beta } + A_1)}, \end{aligned}$$

74 $$\begin{aligned}&A_1 = \hat{\beta }\biggl (-1 + \sqrt{1 + \frac{2}{\hat{\beta }}}\biggr ). \end{aligned}$$

#### Expressions for Antigen Occupancy and Bound Antibody Number

**Fig. 6:**
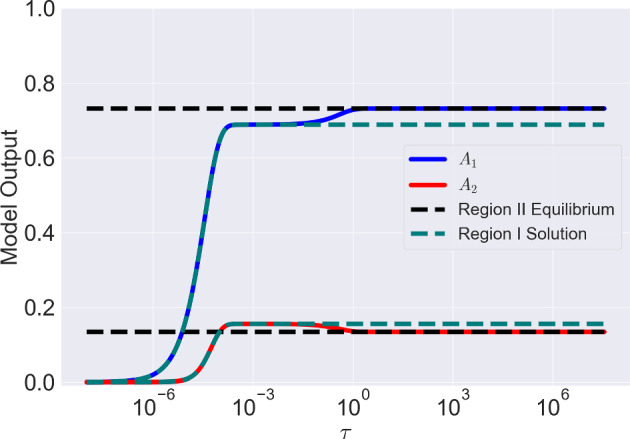
Result of asymptotic analysis of Equations (59) and (60) for $\beta = 10^4$. Region I solutions are obtained by numerically solving Equations (69) and (68). The Region II solution asymptotes for $A_1$ and $A_2$ are obtained from Equations (73) and (74) and are denoted with dashed black lines

From Equations (67), (73) and (74) we have

75 $$\begin{aligned}&A_{occ} = 1 -\frac{\epsilon ^2}{\alpha _1(2\hat{\beta } + A_1)}, \end{aligned}$$

76 $$\begin{aligned}&A_{bound} = \frac{1}{2}\biggl [1+A_1 - \frac{\epsilon ^2}{\alpha _1(2\hat{\beta } + A_1)}\biggr ], \end{aligned}$$

with $\hat{\beta }=\epsilon ^2\beta$ (and therefore $\hat{\beta }$ is $\mathcal {O}(1)$) and $A_1$ given by Equation (74). From Equation (75), as with expressions for $A_{occ}$ for smaller values of $\beta$ with $\beta \ge 1/2$, at leading order antigens are saturated with antibody independent of the model parameters. Therefore, immune checkpoint inhibitors, whose efficacy relies on antigen occupancy, will be insensitive to changes in model parameters in this region of parameter space. Equation (76) at leading order gives $A_{bound}=(1+A_1)/2$ and so, the total number of bound antibodies increases with the number of monovalently bound antibodies as expected. Pertinent for the mechanism of antibodies whose effectiveness depends on the number of bound antibodies, such as in ADCC or for antibody drug conjugates, it follows that the number of bound antibodies can be increased by, for example, increasing the monovalent binding rate, $\alpha _1$, or decreasing $\alpha _2$, the rate at which the second arm bids. Once again, this highlights how avidity may be a barrier for these types of therapeutics. Figure 6 shows that the results of the asymptotic analysis for Region II are in excellent agreement with the numerical solutions of the full system. The bound antibody number when $\beta =\text {ord}(1/\epsilon ^2)$ is larger than the value attained for smaller values of $\beta$, particularly when $\beta =\text {ord}(1)$. Consequently, when $\beta =\text {ord}(1/\epsilon ^2)$ there may be increased mAb therapeutic effects due to increased bound antibody.

## Discussion

Ligand-receptor interactions play a crucial role in drug mechanism of action. Of particular importance are antibody interactions with their cognate antigens. MAbs are antibody-based immunotherapies whose therapeutic effect stems from the dynamics of antibody-antigen binding. In this work, we used asymptotic analysis to identify the key features and parameter dependencies of mAb binding target antigens on a tumour cell surface for a variety of antibody concentrations. This work was motivated by two aims. First we sought to describe and quantify the complex dynamics of bivalent antibody-antigen binding across antibody concentrations commonly seen within in vitro experiments. This was achieved by performing asymptotic analysis for different values of $\beta = A_{\text {tot}}/r_{\text {tot}}$. While many ligand-receptor interactions are monovalent, the binding dynamics of mAbs are more complex due to the bivalency of the antibody. This complexity is introduced through a fast-cross-linking reaction on the cell surface assumed to be driven by the surface diffusion of target antigen as motivated by Sengers et al. (2016); Heirene et al. (2025). Initially, there is a fast transient where the number of monovalent antibodies increases. There is then a short lag before antibodies bind their second arm to form bivalently bound complexes. For values of $\beta =A_{\text {tot}}/r_{\text {tot}}$, that is the ratio of total antibody to receptor, which satisfy $\beta < \text {ord}(\epsilon ^{-2}$) (with $\epsilon = \sqrt{\alpha _1/\alpha _2}$ corresponding to the much faster rate of binding a second antigen on the cell surface compared to the first by an antibody in solution), there are more bivalently bound than monovalently bound antibodies due to the availability of free antigens and the fast surface reaction rate. When $\beta =\text {ord}(\epsilon ^{-2})$, there are enough antibodies competing for free antigen so as to overcome the fast surface reaction rate and, as a result, the number of monovalently bound antibodies is much larger. Through our asymptotic analysis, for the case where antibody is more abundant than antigen, we identified two sub-regions: Region I where free antigen is abundant to bind antibody (also including initial transients of the system) and Region II where levels of free antigen are very small. Analogous results hold for the case where antigen numbers exceed ligand. Within Region II, the system relaxes to a new equilibrium where number of bivalently bound antibodies decreases and the number of monovalently bound antibodies increases. The increase in monovalently bound antibodies further increases the number of bound antibodies. An interesting conclusion can be drawn from the fact the dynamics separate into two regions when antibodies exceed antigens; dissociation events more readily occur when the number of free antigens becomes very small. This is not immediately apparent from Equations (23) and (24) because the dominant process within these equations is the binding of the second arm of the antibody. Therefore, an antibody successfully maintains the dissociation of one of its binding arms away from a bound antigen only when the number of free antigens is small and the binding and dissociation terms are balanced. The second aim of this work has been to use asymptotic analysis to quantify how quantities that correlate with the potency and efficacy of mAb treatment depend explicitly on model parameters. In particular, we are interested in the long time values of antigen occupancy and the number of total bound antibodies (given by $A_1 + 2A_2$, and $A_1 + A_2$ respectively). In our previous work we performed a global parameter sensitivity analysis to explore the general parameter dependencies of these quantities (Heirene et al. 2025). In contrast, here, we used asymptotic analysis to derive explicit expressions for their long-time values for the four considered magnitudes of $\beta$. See Table 3 for a summary of these approximations where a closed form solution was practical.

**Table 3:** Asymptotic approximations for the large time values of antigen occupancy, $A_{occ}$, and bound antibody number, $A_{bound}$. Recall that $\beta = A_{\text {tot}}/r_{\text {tot}}$ is the ratio of total antibody to receptor within the system, $\alpha _1$ and $\alpha _2$ (see Equation (19)) are the nondimensional monovalent and bivalent binding rates respectively with $\alpha _1=\mathcal {O}(1)$, $\alpha _2=\mathcal {O}(\epsilon ^{-2})$ throughout our analysis where $\epsilon = \sqrt{\alpha _1/\alpha _2}$. Below $A_1= \hat{\beta }(-1 + \sqrt{1 + 2/\hat{\beta }})$

Magnitude of $\beta$	$A_{occ}$	*B*
$\beta <1/2, |\beta -1/2|\gg \epsilon ^2$	$2\beta$	$\beta$
$\beta -1/2<\text {ord}(\epsilon ^{2/3})$	$1-\epsilon ^{2/3}\alpha _1^{-2/3} + \text {ord}(\epsilon ^{4/3})$	$\frac{1}{2} - \epsilon ^{2/3}\biggl (\frac{\alpha _1^{-2/3}}{2}\biggr ) + \text {ord}(\epsilon ^{4/3})$
$\beta -1/2=\text {ord}(1)$	$1 - \frac{\epsilon }{\alpha _1}$	$\frac{1}{2} - \epsilon \biggl [\frac{1 - 2\alpha _1(\beta -\frac{1}{2})}{2\alpha _1}\biggr ]$
$\{\text {ord}(\epsilon ^{2/3})<\beta -\frac{1}{2}<\text {ord}(\epsilon ^{-2})|\beta -\frac{1}{2}\ne \text {ord}(1)\}$	$1-\frac{\epsilon ^{5/6}}{\alpha _1\sqrt{\beta -\frac{1}{2}}} + \text {ord}(\epsilon ^{7/6})$	$\frac{1}{2} -\frac{\epsilon ^{5/6}}{2\alpha _1\sqrt{\beta -\frac{1}{2}}} + \text {ord}(\epsilon ^{7/6})$
$\beta =\text {ord}(\epsilon ^{-2})$	$1 -\frac{\epsilon ^2}{\alpha _1(2\hat{\beta } + A_1)}$	$\frac{1}{2}\biggl (1+A_1\biggr ) - \frac{\epsilon ^2}{2\alpha _1(2\hat{\beta } + A_1)}$

The expressions in Table 3 present relationships between model parameters and the quantities of interest depending on the magnitude of $\beta$. These asymptotic approximations can be used for parameter estimation by fitting the above expressions to data and to give intuition on how changing model parameters can impact quantities of interest. In particular, the expressions in Table 3 show that bound antibody number *B* is largest for $\beta =\text {ord}(\epsilon ^{-2})$. This suggests that the only way to enhance monovalent binding and increase the number of bound antibodies, is to have a very high antibody concentration and the marginal impact is small. However, large antibody concentrations may result in higher toxicity for the patient and a balance needs to be determined between maximising bound antibody number and ensuring toxicity is tolerable. Furthermore, antigen occupancy as defined by $A_{occ}$ in Table 3 is shown to be one at leading order once $\beta \ge 1/2$, i.e, antigens are fully saturated. Hence, high levels of antibody do not increase occupancy while a multiple orders of magnitude increase in antibody concentration is required before an increase in bound antibody number is achieved. It is worth noting that the asymptotic analysis shows that model predictions are classified in generality despite the large parameter space. In particular, we have shown there is a simple mechanism that underlies the complex binding behaviours observed. If parameters such as the target antigen density, $r_{\text {tot}}$, were to change, the underlying binding mechanisms would not differ though this would have an impact on the antibody concentration, $A_{\text {init}}$, required to produce the same magnitude of $\beta$. More generally, the results of our asymptotic analysis were in good agreement with the representative numerical simulations. A potential limitation of the model is that we have neglected antigen internalisation. This process typically happens on a longer timescale so may affect the values presented in Table 3. Since we have focused on in vitro antibody binding experiments, which are typically run over an hour or so, the impact of internalisation may be small compared to the in vivo setting (Birtwistle and Kholodenko 2009; Vainshtein et al. 2014; Mazor et al. 2016). While we have taken the behaviour of in vitro assays as indicative of response more generally, the impact of internalisation on much longer timescales merits further study. To summarise, we have presented an analysis of a model of bivalent antibody-antigen binding. Using asymptotic analysis, we have described the complex binding dynamics of bivalent antibody-antigen binding for a wide range of antibody concentrations. For the case of antigen being in excess of antibody, we provide asymptotic approximations to the model simulations that are in good agreement with the numerics. Alternatively, when antibody is in excess of antigen, our analysis shows that the antibody-antigen interactions contain two separate regions. Regions I and II are characterised by an abundance or lack of availability of unbound antigen respectively with Region I additionally containing the initial transients of the system. With the transition from Region I to Region II, the dominant balance within the model equations change and an antibody can dissociate one of its binding arms. We use the results of the asymptotic analysis to derive simple expressions for quantities that impact mAb treatment potency and efficacy such as antigen occupancy, of particular pertinence to immune checkpoint inhibition, and bound antibody number, which in contrast is important for ADCC. Future work could also involve extending our results to the case of bispecific antibodies and binding within the immune synapse.

## Figures and Tables

**Fig. 7:**
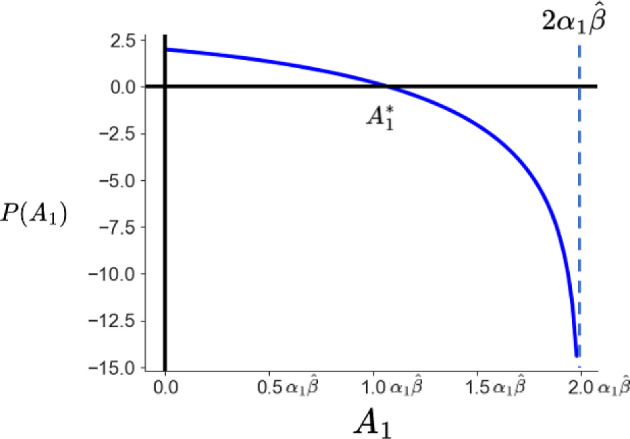
Plot of $P(A_1)$ given by Equation (B9) as $A_1$ varies. The unique root of $P(A_1)$ is denoted $A_1^*$

## Appendix A Justification for magnitude of $\alpha _1$

Throughout the asymptotic analysis, we assume that $\alpha _1 = \text {ord}(1)$. This assumption is motivated by typical parameter values $r_{\text {tot}}=10^5$, $k_{\text {on}}=10^5$ M$^{-1}$s$^{-1}$, $k_{\text {off}}=10^{-4}$ s$^{-1}$ and $k_2=10^{-5}$ s$^{-1}$, for which $\alpha _1$ is indeed order one. However, these parameters, and therefore $\alpha _1$, can vary across several orders of magnitude. To ensure the generality of the asymptotic analysis, we must therefore justify that the relative scaling of terms in Equations (23) and (24) remains consistent even as $\alpha _1$ changes size. To achieve this we need to ensure that there is no jumping of the relative magnitude of terms as $\alpha _1$ varies. In Equations (23) and (24) this corresponds to ensuring

A1 $$\begin{aligned} \epsilon ^2 \ll \alpha _1 \ll \frac{1}{\epsilon }, \end{aligned}$$

noting $\epsilon =\sqrt{\alpha _1/\alpha _2}$, this is equivalent to

A2 $$\begin{aligned} 1 \ll \alpha _2, \end{aligned}$$

and

A3 $$\begin{aligned} \alpha _1^3\ll \alpha _2. \end{aligned}$$

First considering Equation (A2), from the values in Table 1 this is true for all parameter values aside from when the values of $k_2$ and $r_{\text {tot}}$ are very small and the value of $k_{\text {off}}$ is very large. This corresponds to a situation where it is difficult for the antibody to bind its second arm because there are few slowly diffusing antigens and a fast dissociation rate and so the antibody is essentially monovalent. This is situation is outside the aim of this work to study the impact of avidity on bivalent antibody binding dynamics. In the case of Equation (A3), this is equivalent to checking that

A4 $$\begin{aligned} \biggl ( \frac{k_{\text {on}}}{\sigma } \biggr )^3\biggl (\frac{r_{\text {tot}}}{k_{\text {off}}}\biggr )^2 \ll k_2, \end{aligned}$$

which upon substituting values from Table 1 can be verified to be always true. Having established that $\alpha _1$ satisfies Equation (A1), we can conclude that there is no jumping in order as the size of $\alpha _1$ and as a result, $\epsilon$ changes in Equations (23) and (24) and the dominant balances chosen in our analysis hold. Consequently, in the expressions for $A_1$ and $A_2$ obtained throughout our analysis, we can take $\alpha _1$ to be small and the same expressions hold.

## Appendix B Region I limits for $\beta =\text {ord}(1/\epsilon ^2)$

For $\beta =\text {ord}(1/\epsilon ^2)$, the model equations can be cast in terms of $p=2(1-A_1-2A_2)$ and $A_1$:

B5 $$\begin{aligned} \frac{\textrm{d}p}{\textrm{d}\tau ^*}&= -p\biggl (2\alpha _1\hat{\beta } + \frac{A_1}{2}\biggr ), \end{aligned}$$

B6 $$\begin{aligned} \frac{\textrm{d}A_1}{\textrm{d}\tau ^*}&= p\biggl (\alpha _1\hat{\beta } - \frac{A_1}{2}\biggr ), \end{aligned}$$

with initial conditions

B7 $$\begin{aligned} p(0)=2, \end{aligned}$$

B8 $$\begin{aligned} A_1(0)=0. \end{aligned}$$

Dividing Equation (B5) by Equation (B6) and integrating we deduce

B9 $$\begin{aligned} p = 2 + A_1 + 4\alpha _1\hat{\beta }\text {ln}\biggl (1 - \frac{A_1}{2\alpha _1\hat{\beta }}\biggr ) {=}{:}P(A_1). \end{aligned}$$

This expression is valid at small times where $A_1$ is small such that $A_1/2\alpha _1\hat{\beta } <1$. Substituting for *p* in Equation (B6) gives

B10 $$\begin{aligned} \frac{\textrm{d}A_1}{\textrm{d}\bar{\tau }} = \biggl (\alpha _1\hat{\beta }-\frac{A_1}{2}\biggr )\biggl ( 2 + A_1 + 4\alpha _1\hat{\beta }\text {ln}\biggl (1 - \frac{A_1}{2\alpha _1\hat{\beta }}\biggr )\biggr ) {:}{=}F(A_1), \end{aligned}$$

with $A_1(0)=0$. We are interested in the behaviour of $F(A_1)=(\alpha _1\hat{\beta } - A_1/2)P(A_1)=0$ to determine how $A_1$ behaves as $\bar{\tau }\rightarrow \infty$. Let us first look at a graph of $P(A_1)$ in Figure 7.

We can see that *P*(0) = 2 and $P(A_1) \rightarrow - \infty$ as $A_1 \rightarrow 2\alpha _1\hat{\beta }$. Observing Equation (B9) we see that

B11 $$\begin{aligned} \frac{\textrm{d}P}{\textrm{d}A_1} = 1 + \frac{4\alpha _1\hat{\beta }}{A_1-2\alpha _1\hat{\beta }} \le -1, \end{aligned}$$

when $A_1 \in (0, 2\alpha _1\hat{\beta })$. Therefore, $P(A_1)$ and, as a result, $F(A_1)$ has a unique root $A_1^*\in (0, 2\alpha _1\hat{\beta })$ with $A_1\rightarrow A_1^*$ as $\bar{\tau } \rightarrow \infty$. Therefore, $P(A_1) \rightarrow 0$ as $\bar{\tau } \rightarrow \infty$. But $p = P(A_1)$ so therefore $p \rightarrow 0$ as $\bar{\tau } \rightarrow \infty$. From this

B12 $$\begin{aligned} p \rightarrow 0 \text { as } \tau \rightarrow \infty . \end{aligned}$$

Finally, as a result we have

B13 $$\begin{aligned} A_1+2A_2 \rightarrow 1 \text { as } \tau \rightarrow \infty . \end{aligned}$$

## Declarations

### Code availability

All model simulations were conducted in Python with scripts available by contacting luke.heirene@maths.ox.ac.uk.
